# The COVID-19 Outbreak and Affected Countries Stock Markets Response

**DOI:** 10.3390/ijerph17082800

**Published:** 2020-04-18

**Authors:** HaiYue Liu, Aqsa Manzoor, CangYu Wang, Lei Zhang, Zaira Manzoor

**Affiliations:** 1 Business School, Sichuan University, Chengdu 610064, China, seamoon@scu.edu.cn (H.L.); 2017141081028@stu.scu.edu.cn (C.W.); 2 School of Computer Science, Sichuan University, Chengdu 610064, China; 3 Economics Department, Shandong University, Jinan 250100, China; zairamanzoor@yahoo.com

**Keywords:** COVID-19, investor sentiment, abnormal returns, stock market indices

## Abstract

This paper evaluates the short-term impact of the coronavirus outbreak on 21 leading stock market indices in major affected countries including Japan, Korea, Singapore, the USA, Germany, Italy, and the UK etc. The consequences of infectious disease are considerable and have been directly affecting stock markets worldwide. Using an event study method, our results indicate that the stock markets in major affected countries and areas fell quickly after the virus outbreak. Countries in Asia experienced more negative abnormal returns as compared to other countries. Further panel fixed effect regressions also support the adverse effect of COVID-19 confirmed cases on stock indices abnormal returns through an effective channel by adding up investors’ pessimistic sentiment on future returns and fears of uncertainties.

## 1. Introduction

**Figure 1:**
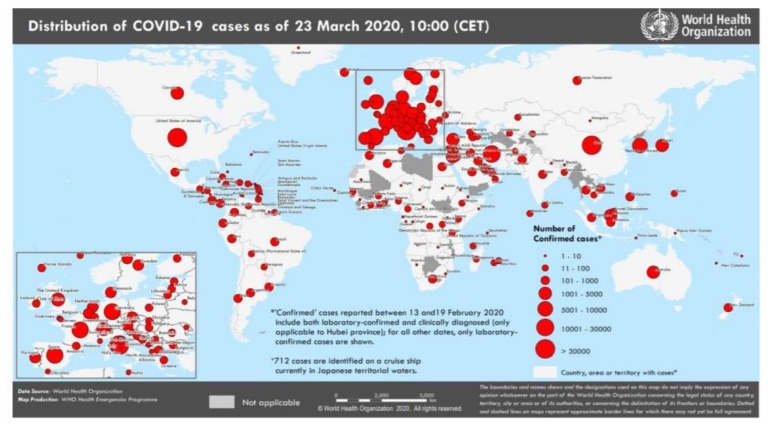
World Health Organization (WHO) situation report 63 (https://www.adb.org/publications/economic-impact-covid19-developing-asia).

On 31st December 2019, the World Health Organization (WHO) identified the first case of COVID-19 in Wuhan China (https://www.who.int/emergencies/diseases/novel-coronavirus-2019). In early and mid-January 2020, the virus started to spread to other Chinese provinces, supported by a huge movement of people towards their hometowns to celebrate Chinese New Year which turned the outbreak into a national crisis. Although Wuhan officials announced a complete travel ban in terms of its residents on January 23, the virus still spread quickly. The WHO declared a global emergency due to the rapidly spreading of COVID-19 on January 30, 2020. It’s only the sixth time that such type of global emergency has been announced, with past examples including that of the Democratic Republic of Congo Ebola outbreak and the Zika virus. Chinese scientists linked this disease to a virus family known as coronaviruses, which includes both the severe acute respiratory syndrome (SARS) virus and the Middle East respiratory syndrome (MERS). According to the Centre of Disease Control and Prevention (CDC), the COVID-19 symptoms may occur within as few as 2 days or as long as 14 days after exposure or contact with an already affected person, which makes it even harder to confirm and control during early stages. By assessing the risk of spread and severity of COVID-19 outside China WHO declared this virus as a pandemic on March 11, 2020. The fatality rate of COVID-19 as compare to other known viruses is quite low, but its infection rate is relatively high (Table 1). As of March 23, China, Italy, and the United States have most of the number of confirmed cases of COVID 19,81601, 59,138, and 31,573 respectively (WHO situation report–63, Figure 1). According to CDC and many other researchers at the moment, the source of COVID-19 is unknown and there is no specific vaccine and treatment [1,2,3]. 

The WHO and public health officials performed the role of mediator to communicate the risk of an outbreak to the investors and it shapes the investors’ sentiments towards the disease [4]. Investor’s sentiments influence the stock markets significantly. When the market is trending upwards and there is less perceived risk then investor behaves more optimistically. When the market is trending downwards then investors’ sentiments become relatively pessimistic and investors will tend to wait to enter the market until a revival begins [5,6]. Such situations lead to short term investor overreaction. Shu [7] studied how mood affects financial market behavior. The study shows how the fluctuations in investor mood directly affect prices for equilibrium assets and projected returns. Researchers suggest that media coverage also affects the actions of investors, the higher the number of articles relating to unexpected events, the greater the number of withdrawals [8,9,10]. Globalization has linked economies worldwide and increased the interdependence of global financial markets in recent years. This increased interdependence among the global stock markets may have an impact on global investors’ decisions on asset allocation and on economies as well as economic policies to ensure economic stability [11]. By using a vector auto regression model, In, Kim, and Yoon [12] examined the dynamic linkages and interactions between the Asian stock markets and their results showed that the markets became more closely linked during the financial crisis, except Malaysia. For any global financial market analyst, it is obvious that stock markets continue to move in the same direction in different countries. There are some variations, however, in the sense that some stock markets appear more correlated with each other than others [13]. Although globalization brings many significant economic advantages, it also plays an important role during infectious global crises [14]. The planet has recently been hit by increasing numbers of infectious diseases such as Crimean Congo hemorrhagic fever, Ebola virus, MERS CoV, SARS, Lassa fever, Nipah Virus, avian flu, Rift Valley fever, Zika virus. The spread of contagious disease not only affects people’s health and lives but also induces a decline in economic growth. 

Explaining why market participants make decisions contrary to rational market participants’ assumptions is one of the central issues in the behavioral finance studies. There are major challenges of COVID-19 to personal lives, including lockdowns (or lockdown-like situations) for a large number of people. Besides the extreme occurrences of death and disease, many people across the globe are panicking because of this fast-spreading infectious disease. Such external and unexpected shocks can bring down economic trends and suddenly change investor’s sentiments. Kaplanski and Levy [15] suggest that investment decisions can be affected by bad mood and anxiety and that anxious individuals may be more pessimistic about future returns and therefore tend to take fewer risks. Anxiety creates a negative feeling which can impact investment decisions and the subsequent returns on assets.

The unusual situation developed by COVID-19 offers us an opportunity to assess the pandemic’s impact on the stock markets of affected nations due to an unforeseen and feared disease. In this paper, we discuss the effect of COVID-19 on major affected countries’ stock markets as measured by their leading stock indices in Japan, Singapore, Korea, Thailand, Indonesia, Russia, Malaysia, the USA and Germany, etc. Due to the short time of the virus outbreak, an event study is conducted to examine the impact of the unexpected outbreak of COVID-19 on stock market indices performances. 

The remainder of the paper is organized in the following sections: Section 2 includes the related theoretical and empirical literature, the data and methodology are discussed in Section 3, followed by the empirical evidence in Section 4, and Section 5 includes a conclusion.

## 2. Literature Review

The impact of the COVID-19 is of crucial importance, especially since its first outbreak happened in China, which is the main hub of foreign investment in Asia. Researchers believe that COVID-19 and SARS belong to the same family, but these two epidemics differ significantly. Many previous studies related to the economic effects of the infectious virus epidemic could be referred to as we discuss the impact of COVID-19.

### 2.1. Economic Impact of Virus Outbreak

Existing literature concentrates on illness-associated costs of medical or economic effects arising from morbidity as well as mortality due to disease. Siu and Wong [16] studied the spread of Hong Kong’s SARS epidemic, and addressed its economic impact and suggested that the most serious negative impacts were seen on the consumer side, with the short term severely affected by local consumption and the export of tourism and air travel-related services. The economy did not face any supply shock, as the manufacturing base present in the Delta of the Pearl River was unaffected and products were usually exported to Hong Kong. By using the G-Cubed (Asia Pacific) model Lee and McKibbin [14] evaluated the global economic impacts of the severe acute respiratory syndrome (SARS) and according to them the effect of the SARS epidemic on human society all over the world is severe, not only because the disease spreads rapidly through countries by global travel, but also because of financial integration and globalization, any economic shock to one country spreads rapidly to others. Ichev and Marinč [17] investigated whether the geographical proximity of information disseminated by the 2014 Ebola outbreak, coupled with widespread media coverage, has affected US asset prices. The results show that the effect on stock prices is generally negative, while local media reporting also has a significant impact on local trading, and the effect is more pronounced in smaller and more volatile stocks and less stable industries.

### 2.2. Impacts on Stock Market Performances

Looking at the effect on stock markets, DeLisle [18] proposed that the cost of the 2003 SARS outbreak resulted in losses as high as in the financial crisis of Asia, estimated at $3 trillion value in GDP and $2 trillion value in financial markets equity. Nippani and Washer [19] examined the effect of SARS on Canada, China, the particular administrative region of Hong Kong, Indonesia, China, Singapore, the Philippines, Vietnam and Thailand and concluded that SARS only affected the stock markets of China and Vietnam. Del and Paltrinieri [9] evaluated the 78 mutual equity funds geographically based in African countries with observed monthly flows and results for the 2006–2015 period and suggested that Ebola and the Arab Spring seriously affect the funds flows, controlling the performance of the funds, spending, and returns of the market. Macciocchi et al., [20] studied the short-term economic impact of the Zika virus outbreak on Brazil, Argentina and Mexico, and their results showed that, with the exception of Brazil, the market indices of these three Latin American and Caribbean Countries (LCR) did not show large negative returns the day after each shock. The average return was −0.90 percent but on different occasions and countries it ranged from 0.90 percent to −4.87 percent. Ming-Hsiang Chen, Shawn, and Gon [21] checked the SARS outbreak impacts on the efficiency of Taiwanese hotel stocks using an event study approach and found that during the SARS outbreak period, seven publicly traded hotel companies experienced steep declines in income and stock price. Taiwanese hotel stocks showed significant negative cumulative mean abnormal returns on and after the day of the SARS outbreak, indicating a significant impact of the SARS outbreak on performance in hotel stock. Mei-ping Chenet al., [22] analyzed the effect of the SARS epidemic on China’s long-term relationship with four Asian stock markets their findings support the existence of a time-varying co-integration relationship in aggregate stock price indices, and they also found that the SARS epidemic has weakened China’s long-term relationship with the four markets. Wang, Yang, and Chen [23] suggested that infectious disease outbreaks have a major impact on the performance of biotechnology stock in Taiwan. According to Bai [24] Baker, Wurgler, and Yuan [25] investors may feel pessimistic about investment prospects in a given market, selling off that market’s stocks under communicable disease outbreak.

### 2.3. Linkages between Stock Markets during Crisis

Stocks markets are interlinked and interdependent. Researchers have discovered the close cross-market correlations during the crisis. Chiang, Nam, and Li [26] examined the daily stock return for nine Asian markets for the period of 1996 to 2003 and found that there was a high correlation among sample Asian countries during the period of crises. Sun and Hou [27] found that in Southeast Asia, Malaysia, Vietnam, and Thailand were most financially integrated with China. According to Morales and Callaghan [28] the global stock markets were becoming more interdependent and crisis in one country would soon spread to another. Stock market movements become increasingly correlated. Events like infectious disease outbreaks can induce negative changes in investors’ sentiment that strongly affects their investment decisions and, consequently, stock market prices. In countries that are culturally more susceptible to herd-like actions and overreaction or countries with low institutional participation, the effect of investor sentiment on stock markets is more pronounced [29,30]. 

## 3. Event Study Method

Mackinlay [31] believed that the idea of event study method was first embodied in research by Dolley [32] before that Ball and Brown [33] and Famaet al., [34] first proposed the method systematically. According to the theory of the event study method, when an efficient market hypothesis is valid, the influence of a particular event will be reflected in the change of stock price, to explain the effect on the return of stocks and reaction to information disclosure. Therefore, the event study method is widely used in economics and finance empirical studies to identify the impact of specific events. For example, Agrawa and Kamakura [35] studied the effect of celebrity endorsement through the analysis of abnormal stock returns. Gaver, K. M., and Battistel [36] studied stock market responses to the adoption of long-term compensation agreements for top management. Thompson [37] analyzed the impact of anticipated sectoral adjustments to the Canada–United States Free Trade Agreement on industry-level stock returns and proposed that the overall impact of trade liberalization on the economy was positive. Additionally, other studies on the impact of sudden diseases on the stock market have applied the event study method as well.

Wang et al., [23] investigated how outbreaks of infectious diseases affected the performance of biotechnology stocks, showing that Taiwan’s biotechnology industry had significant abnormal returns due to statutory infectious diseases.

Based on existing literature, event study methodology is chosen to investigate the abnormal returns (ARs) and cumulative abnormal returns (CARs) of the leading stock indices of affected countries under the COVID-19 outbreak.

### 3.1. Data and Methodology

#### 3.1.1. Data of the Selected Stock Indices and Benchmark Index for Estimation

The following 21 stock indices in Table 2, which are the most representative indices of the stock markets in affected countries and areas, were chosen to assess the impact of the COVID-19 outbreak.

Dow Jones Global Index, an international index reflecting the overall performance of stock markets across the world, is selected as the benchmark index to calculate the abnormal returns of composite indexes listed above. We collected daily closing prices of these indexes from 21 February, 2019 to 18 March, 2020. The data sources used for this study are the China Stock Market & Accounting Research (CSMAR) database and website Investing.com (a website offering free real time quotes, portfolio, streaming charts, live stock market data, etc.).

#### 3.1.2. Event Study Set-up

In this paper, we examine the impact of the unexpected outbreak of COVID-19 on stock markets of affected countries. According to several COVID-19 news sources, in late December 2019 a new disease outbreak was recorded in Wuhan. Later, on Dec. 31, the virus was first identified to the WHO. But it was not until 20 January, 2020, when the National Health and Fitness Commission of the People’s Republic of China high-level expert group leader Zhong Nanshan proposed in an interview that the new coronavirus could be transmitted among people, that the disease attracted wide public attention. Right after the interview, the infectious coronavirus began to appear in the press over the world, which grabbed the headlines of the major media. Thus, 20 January, 2020, when the news broke out causing a stir, is selected as the event day. To study the influence in different periods, we set up five event windows consisting of 35 trading days after the event day: (0, 6), (7, 13), (14, 20), (21, 27), (28, 34). Referring to related researches [23,38], we define the estimated window of 90 trading days before the event day when studying the influence of infectious diseases on the market behavior. As there is a lot of uncertainty in the stock market, too long a window period may not be accurate. To test the sensitivity of our results, we also use (‒1,–120), (–1,–150) and (–1,–180) as the estimated windows to compute the abnormal returns. We use the T-test to test the significance of the results and change the event window and estimated window to strengthen the robustness. Moreover, results from event windows of different lengths reflect the various response speeds and changing trends of the stock market. The expected returns are derived using the market model, and the ordinary least square (OLS) based on the following regression model:

(1) $$R_{i,t}=\alpha_{i}+\beta_{i}R_{mt}+\varepsilon_{i,t}$$

*R_i,t_* is the return of index i and *R_mt_* is the market return on day t (as the event day is day 0) within the estimated window, with $\varepsilon_{i,t}$ as the statistic disturbance. After obtaining the estimated coefficients, $\hat{\alpha_{i}}$ and $\hat{\beta_{i}}$, the following formulas are applied to calculate the expected return and abnormal return (AR):

(2) $$E\left(R_{i,t}\right)=\hat{\alpha_{i}}+\hat{\beta_{i}}R_{mt}$$

(3) $$AR_{i,t}=R_{i,t}-E\left(R_{i,t}\right)$$

*E*(*R_i,t_*), *R_i,t_* and *AR_i,t_* are the expected return, real return and abnormal return of index i on day t within the event window. The average abnormal return of sample indices on day t is calculated as:

(4) $$AAR_{t}=\frac{1}{N}\overset{N}{\underset{i=1}{\sum }}AR_{i,t}$$

where t = (0,1,2…32,33,34), and N is the total number of observations. Abnormal return and average abnormal return can be accumulated over time. Cumulative abnormal return (CAR) of index i over a while from t_0_ to t_1_ and cumulative average abnormal return (CAAR) are calculated based on Equations (5) and (6):

(5) $$CAR_{i}\left(t_{0},t_{1}\right)=\overset{t_{1}}{\underset{t=t_{0}}{\sum }}AR_{i,t}$$

(6) $$CAAR\left(t_{0},t_{1}\right)=\overset{t_{1}}{\underset{t=t_{0}}{\sum }}AAR_{t}\;$$

## 4. Empirical Results of Event Study on AR and CAR

The mean and standard deviation of the composite index return before and after the event are given in Table 3. As the basic statistic description, where Panel A shows the data from 21 February, 2019 to 19 January, 2020 and Panel B shows the data from 20 January, 2020 to 18 March, 2020, Table 3 indicates that after 20 January, 2020, all the mean returns decreased and most standard deviations increased compared with the previous ones. The indices for France, Germany, Russia, Italy, Thailand, the UK, Canada, Japan, the USA, India, Abu Dhabi and Australia decreased the most in mean return, by 0.01 approximately, while those for Singapore, Thailand, Korea, Indonesia, and Hong Kong fell the most (by 325.245%, 274.619%,115.163%, 64.345%, and 49.086%, respectively) by percentage. Whereas, the mean returns of SSEC and SZCS, which represent the market of the mainland of China, fell the least in percentage. It appears that COVID-19 reduces the stock market returns in all affected countries and increases their volatility, showing not only a greater impact on the stock markets in Asia but also an inescapable influence on those in countries out of Asia.

Table 4 illustrates the ARs of the sample indices on and after 20 January, 2020. On the day of the event, the representative composite indices for France, London, Malaysia, Indonesia, Hong Kong, Singapore, Thailand, Italy and India react most rapidly with negative ARs. On the following day, ARs of ADX, DJIA, FTSE100, KOSPI, IMOEX.ME, N225, AXJO, STI, TPE TAIEX, AAXJ, SET50, HSI, SSEC, SZCS, FTMIB and NSEI are negative. It can be seen that the actual returns of Asian countries were further away from the expectation than that of other regions, with indices representing markets in Taiwan, Hong Kong, Shanghai and Shenzhen indexes decreasing most significantly on day 1. It indicates that the Chinese stock market suffered a serious negative impact when the news of the coronavirus was firstly widely reported by the international media.

**Figure 2:**
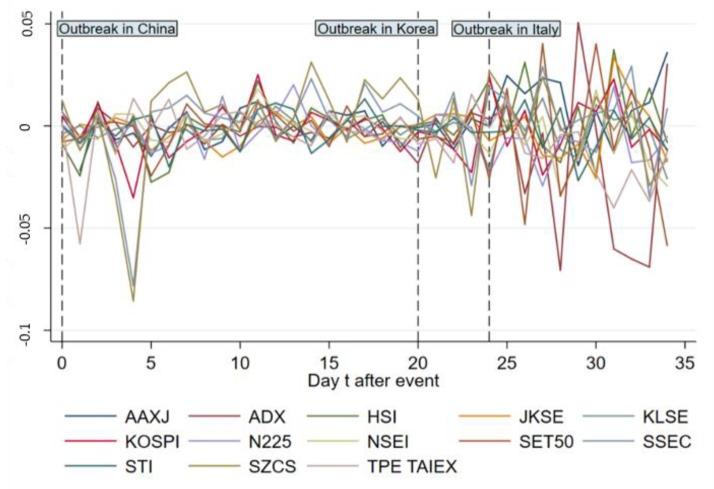
Abnormal Return (AR) change of main indices in Asia from day 0 to 34.

**Figure 3:**
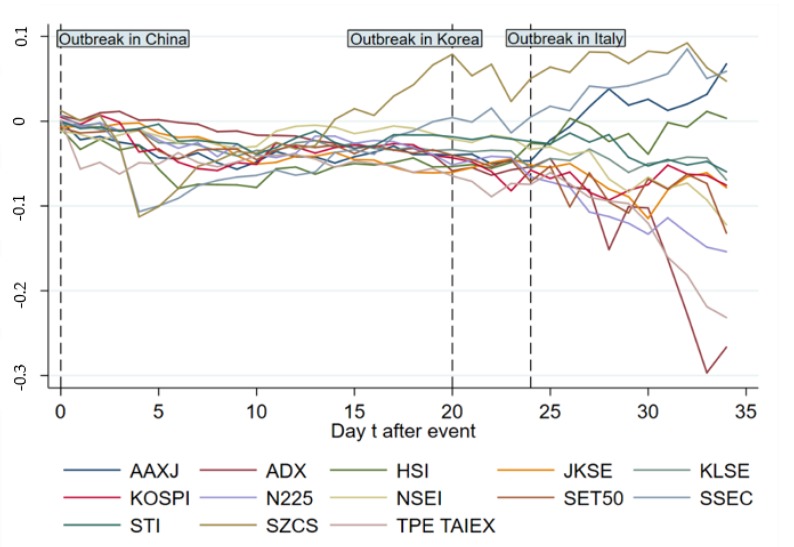
Cumulative abnormal return (CAR) change of main indices in Asia from day 0 to 34.

Figure 2 and Figure 3 give ARs and CARs of the main indices in Asia from day 0 to 34, showing that most ARs become negative during 1 day after the event. The pandemic broke out in Korea and Italy on 19 February, 2020 and 21 February, 2020, respectively, indicating another two big events of COVID-19; we marked the two outbreaks in the figures to show the specific reaction of stock markets on the timeline. In Figure 2, some indices for instance, TPE TAIEX, HSI and SZCS, saw a sharp decline in AR right after the event day. On day 4, ARs of Asian main indices experience a dramatic fall with the biggest drop in SZCS for Shenzhen, SSEC for Shanghai and KOSPI for Korea, rendering the following fluctuation, which becomes more violent after day 24 (outbreak in Italy). Figure 3 shows that the CARs of included indices keep going down overall from day 0 to 4, after which SZCS and SSEC keep going up from day 5 to 20. The following violent fluctuation of ARs has different cumulative effects on indices as CARs of SZCS, SSEC, HSI and AAXJ increase in general while others decrease or stay stuck. 

**Figure 4:**
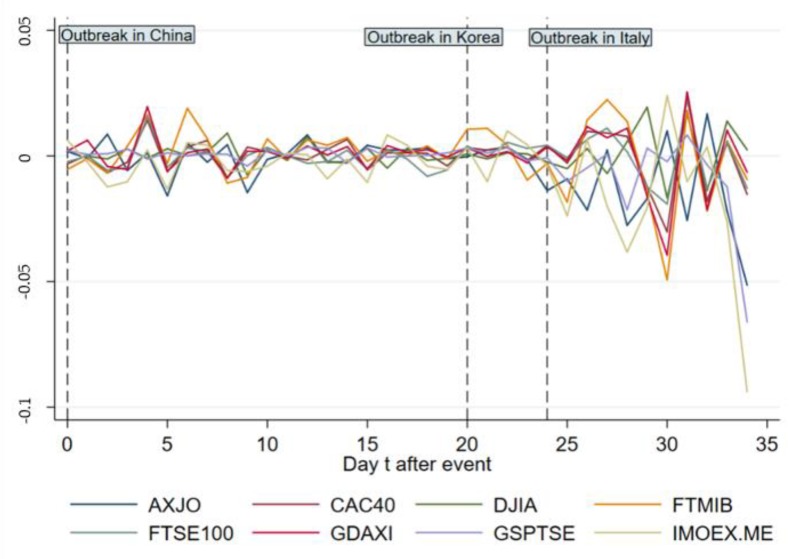
AR change of main indices out of Asia from day 0 to 34.

**Figure 5:**
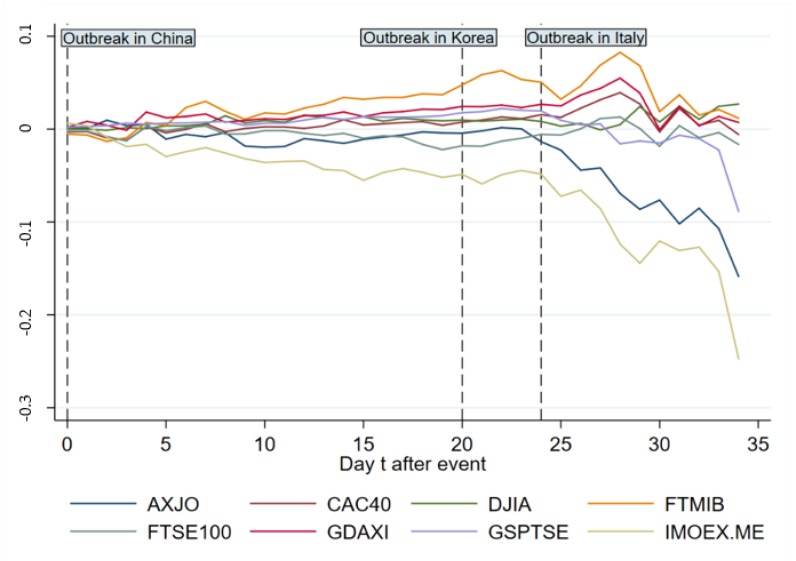
CAR change of main indices out of Asia from day 0 to 34.

Figure 4 and Figure 5 exhibit ARs and CARs of main indices out of Asia from day 0 to 34. As is shown in Figure 4, the ARs witness violent fluctuations relatively except those of GSPTSE for Canada and DJIA for America, with a drastic “up and down” between day 3 and day 5. After day 24, a violent fluctuation occurs across all indices showing an obvious negative influence on ARs. Figure 5 indicates that before day 24 there is no significant effect on CARs except a gentle decline and increase for IMOEX.ME and FTMIB respectively. After day 24, CARs of most indices decrease in general.

Table 5, Table 6, Table 7, Table 8 and Table 9 compare the significant CARs of affected countries in different event windows. Table 5 illustrates that in the event window (0, 6), indices for Hong Kong, Malaysia, Japan, Thailand and Asia ex-Japan show significant negative CARs while Canada shows a significantly positive CAR. It appears that Asian countries experience an obvious downturn after the breakout of COVID-19 immediately. According to the data shown in Table 6, the CAR of Abu Dhabi representative index in the event window (7, 13) is –0.021254 (5% level), while those of Shanghai and Shenzhen representative index turn significantly positive at 0.0311078 (10% level) and 0.0506241 (10% level), respectively, which demonstrates the recovery of the Chinese stock market as in China the spread of COVID-19 is being controlled.

Table 7 shows that in the event window (14, 20), N225 for Japan and ADX for Abu Dhabi show significant negative CARs at –0.0337579 (5% level) and –0.0356547 (10% level), respectively. SZCS for Shenzhen and SSEC for Shanghai are still significantly positive in CARs. The effects of COVID-19 on stock markets during this period are not as significant as a whole. Table 8 indicates the indices for the UK and France show positive CARs at 0.029436 (1% level) and 0.0241526 (10% level), respectively. During this time window, Europe has not become the center of the pandemic outbreak. Table 9 shows that in the window (28, 34), CARs of indices representing Taiwan, India and Australia are –0.1419123 (1% level), –0.0873618 (10% level) and –0.1167886 (10% level). Most indices of countries out of Asia for instance, France, the UK, Russia and Italy, have negative CARs.

The results show that the stock markets in Asia, especially in Hong Kong, Malaysia, Japan, and Thailand, responded rapidly to the news of the coronavirus outbreak. For the mainland Chinese market, the negative influence does not last for long as SZCS and SSEC show significantly positive CARs in the event window (7, 13) and (14, 20). This demonstrates the quick recovery of the mainland Chinese market from the pandemic after the confirmed cases decrease. For stock markets in countries out of Asia, there is no noticeable decline of cumulative abnormal return until day 24 in this group causing negative CARs for most countries, especially significant for Australia.

**Figure 6:**
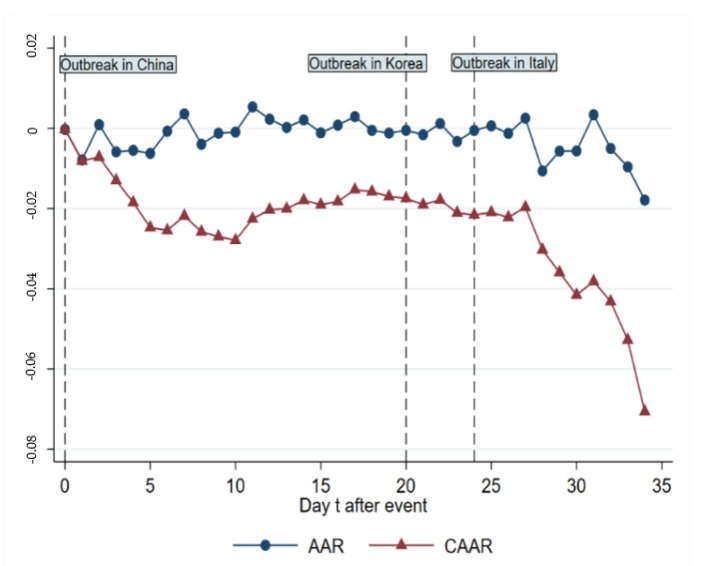
Average abnormal return (AAR) and cumulative average abnormal return (CAAR) change from day 0 to 34.

The results of the daily cumulative average abnormal return (CAAR) across all indices, which is using an average of 21 indices we chose, are shown in Table 10, indicating that most of the CAARs are significant and decrease over time, from –0.0002348 on day 0 to –0.0706297 (5% level) on day 34. Figure 6 illustrates the change of AAR (average abnormal returns of all indices) and CAAR from day 0 to 34, which is a downward sloping trend as a whole with stagnation in between day 10 and day 27. It seems that there are two plunges in stock markets on day 1 and day 24, which roughly match the outbreaks in and out of Asia. Similar results using (–1, –120), (–1, –150) and (–1, –180) as the estimated windows also support the findings which shows the robustness of event negative effect on AR and CAR using (–1,–90) as estimation window (available under inquiry).

To test for possible COVID-19 outbreak effects and transmission channel on major stock market indices we used panel data for 21 market indices in a 35-day window after the outbreak. We conducted ordinary least square (OLS) regressions to analyze the outbreak effect. The variables we chose to include in the empirical model are discussed in the earlier sections as daily abnormal returns (*AR*) as a dependent variable (the calculation of AR is using Equation (3) in the earlier section: event study set-up). The independent variable is the logged global COVID-19 confirmed cases (*Log_case*) which we extracted from the WIND database. Furthermore, we controlled global market systematic risks using Dow Jones Global Index daily returns (*ReturnM*) and country specific systematic risks using daily returns of each index (*Return*). In further regressions to test the mediating effect, we used S&P 500 volatility index (VIX) provided by the Chicago Board Options Exchange which was widely used as a proxy to gauge investors’ fear. The summary statistics are shown in Table 11.

It can be seen that the mean of *AR*, *ReturnM*, and *Return* are all negative after the virus outbreak.

## 5. OLS Regression Results of COVID-19 Confirmed Cases and Stock Market Indices ARs

A panel data analysis was conducted to capture the major stock market indices performance after the outbreak. The panel dataset consisted of a cross-section dimension (21 indices, i = 1, …N) and a time dimension (35 periods: t = (0, …34)), there were over 735 observations, which was considered adequate to produce robust estimations within the scope of the analysis. To begin, we used OLS to analyze the country-level stock market indices in response to the virus outbreak while controlling for global and country-specific characteristics embedded in stock indices, including country and year fixed effects. In our main tests, we analyze how the confirmed cases of COVID-19 affects the abnormal return of major markets stock indices. Then we set the dummy variable *Asia* to group the market indices to see the regional effect of the virus outbreak as it could be spreading faster or getting more attention in Asian areas at the beginning of the outbreak in our study period. If the index belongs to the country in Asia, *Asia* equals to 1, otherwise, *Asia* equals to 0. The following is the baseline model of our regression:

(7) $$AR_{it}=\alpha +\beta_{1}Log\_case_{it}+\beta_{2}Return_{it}+\beta_{3}ReturnM_{it}+\gamma_{i}+\delta_{t}+\varepsilon_{it}$$

First, we run regression on *Log_case* alone and we gradually add *Return*, *ReturnM*, before at last we add dummy variable *Asia* to test the regional effect. The mean-centered variance inflation factors (VIFs) for the independent variables specified are 3.01 which means there is no collinearity issue in the regression model. We report robust standard errors to deal with the heterogeneity problems that is always a concern with economic data. The results in Table 12 show a significant negative relationship between confirmed COVID cases and stock market indices daily abnormal returns after the outbreak. *Asia* gets a negative sign which indicates indices in Asian areas as a whole suffered more in their performances compared with those which are out of the region. These results also echo our earlier analysis using the event method. As a robustness check of our ordinary least squares (OLS) regression we used feasible generalized least squares (FGLS) estimation with heteroscedastic error terms including time dummies. The results also show the negative significance of COVID-19 confirmed cases on *AR* (available under inquiry).

## 6. Transmission Channel of COVID-19 Outbreak on Stock Market Indices

The public health emergency could transmit the effect to the economy as the stock market serves as the barometer of investors’ expectations and faith in economic prospects [24,25]. COVID-19 pandemic compounds uncertainties worldwide, increases stock investors’ fear and creates pessimistic sentiments on future returns. To study the channel by which COVID-19 transmits the fear to stock markets globally, we conducted further regressions to test the mediating effect through the channel of VIX. We set paths to test models as follows (Path A: equation (8), Path B: equation (9), Path C: equation (10)):

(8) $$AR_{it}=\alpha +\beta_{1}Log\_case_{it}+\beta_{2}Controls_{it}+\gamma_{i}+\delta_{t}+\varepsilon_{it}$$

(9) $$VIX_{it}=\alpha +\beta_{1}Log\_case_{it}+\gamma_{i}+\delta_{t}+\varepsilon_{it}$$

(10) $$AR_{it}=\alpha +\beta_{1}Log\_case_{it}+\beta_{2}VIX_{it}+\beta_{3}Controls_{it}+\gamma_{i}+\delta_{t}+\varepsilon_{it}$$

According to Baron and Kenny [39], if $\beta_{1}$ in front of *Log_case* in path A and $\beta_{1}$ in front of *VIX* in path B both show significance, while $\beta_{1}$ is insignificant and $\beta_{2}$ is significant in path C, then we could claim that VIX is an effective mediator between confirmed cases and stock indices AR. The results in Table 13 indicate the mediator variable *VIX* is positively correlated to *Log_case* with significance in columns 2 and 3 (Table 13). In column 3, *Log_case* is not significant after adding *VIX* into the regression. VIX is indeed a complete mediator and the fears caused by the COVID-19 pandemic transmitted to the stock markets by the channel of cumulated panic and uncertainties.

## 7. Conclusions

This research has aimed to analyze the immediate effect of COVID-19 on the stock markets of the major affected countries. This research adds to the literature as it explores the unexpected outbreak effects on financial markets of a feared disease. From the viewpoint of an investor, the findings of this analysis illustrate the importance of not only the company’s business factors but also the investment risks brought on by such a sudden event. Our results suggest: (1) COVID-19 outbreak has a significant negative effect on stock market returns across all affected countries and areas. Two plunges in stock markets AAR and CAAR on day 1 and day 24 match the outbreaks in and out of Asia. (2) Stock markets of Asian countries react more quickly to the outbreak with some of them recovering slightly in the later stage of the pandemic. (3) Confirmed cases of COVID-19 have significant adverse effects on major stock indices performances with those in Asia suffering a greater decrease in terms of abnormal returns. (4) Investor’s fear sentiment is proved to be a complete mediator and transmission channel for the COVID-19 outbreak’s effect on stock markets.

As the COVID-19 epidemic now becomes a pandemic, we need to think of not only ways to avoid future public health problems but also financial issues as well. The virus spreads exponentially, doubling new infections every two to three days, or even quicker. Fears of pandemic and policy measures to control disease transmission have contributed to a global supply shock, especially in the labor-intensive and manufacturing sector. To safeguard the staff, factories and offices are shutting or reducing activities which decreases labor force, productivity, and ultimately affects the profitability of companies. It would leave several businesses illiquid and, if not handled correctly by officials, would cause companies to resort to staff cutbacks or to shut down entirely. This is the main explanation of why financial markets have been in panic mode worldwide. Stock prices represent the potential of future earnings, and investors see the pandemic as a dampening economic activity and are concerned about future revenue. Before the severity of the deterioration is evident, the normal investors’ response would be to sell the stocks. 

Our findings have significant implications for policymakers. A coalition of government officials, investment banks regulators, and the central bank would be required to tackle this challenge. Through rolling over current loans, bank authorities would allow banks to be lenient towards businesses in badly impaired economic sectors such as manufacturing, travel and tourism. Managing the COVID-19 crisis needs a rational approach such that officials should immediately inform citizens of what they and the health care system will do without triggering uncertainty.

This paper presents an initial analysis of the pandemic issue; there is significant room for further research into investor confidence inside and between foreign markets. In future studies, the research could be taken on investor sentiment and uncertainty as a framework. Considering the practicality of our conclusions, we conclude that our results would be valuable for institutional and individual investors, fund managers, financial, industrial analysts, and public health officials to effectively communicate the risk of an infectious disease. Health officials must consider the psychological and sentimental impact of their announcements as well.

As with all studies our work has several limitations, one of them is that we only studied the immediate and short-term effects of COVID-19 on majors affected countries’ stock markets due to the short event window period and the evolving nature of the virus spread. Another limitation is that we didn’t study the demographic variables such as age, gender, education level, experience in the stock market and type of investor, etc. due to lack of data.

## Figures and Tables

**Table 1:** Fatality Rates and Infection Rates of COVID-19 and Other Epidemics.

Epidemics	Fatality Rate (Deaths/Cases)	Infection Rate (Per Infected Person)
Ebola	50%	1.5–2.5
MERS	34.30%	0.42–0.92
SARS	10%	3
COVID-19	1%–3.4%	1.5–3.5
Seasonal flu	1%–3.4%	1.3

Source: Asian development bank report No. 128(https://www.adb.org/publications/economic-impact-covid19-developing-asia).

**Table 2:** Selected indices for affected countries and areas.

Definition	Abbreviation	Country/Area
Abu Dhabi Securities Exchange (ADX) Composite Index	ADX	Abu Dhabi
Cotation Assistée en Continu (CAC) 40 Index	CAC40	France
Deutsche Aktien Xchange (DAX) Performance Index	GDAXI	Germany
Dow Jones Industrial Average Index	DJIA	The USA
Financial Times Stock Exchange (FTSE) 100 Index	FTSE100	The UK
FTSE Bursa Malaysia Kuala Lumpur Composite (KLCI) Index	KLSE	Malaysia
Jakarta Composite Index	JKSE	Indonesia
Korea Composite Stock Price Index	KOSPI	Korea
Moscow Exchange (MOEX) Russia Index	IMOEX.ME	Russia
Nikkei 225 Index	N225	Japan
S&P/Australian Securities Exchange (S&P/ASX) 200 Index	AXJO	Australia
S&P/Toronto Stock Exchange Composite Index (S&P/TSX) Composite Index	GSPTSE	Canada
Straits Time Index	STI	Singapore
Taipei (TPE), Taiwan Stock Exchange (TAIEX) Index	TPE TAIEX	Taiwan
iShares Morgan Stanley Capital International (MSCI) All Country Asia ex Japan Exchange Traded Fund (ETF)	AAXJ	Asia ex Japan
Stock Exchange of Thailand (SET) 50 Index	SET50	Thailand
HangSeng Index	HSI	Hong Kong
Shanghai Composite Index	SSEC	Shanghai
Shenzhen Composite Index	SZCS	Shenzhen
FTSE Milano Indice di Borsa (MIB) Index	FTMIB	Italy
National Stock Exchange (NIFTY) 50 Index	NSEI	India

**Table 3:** Differences in mean returns of sample indices.

Index	Number of Trading Days	Event Group’s Mean	Event Group’s Std. Dev.
Panel A: Pre-event period from 2-21-2019 to 1-19-2020			
AAXJ	230	0.0004542	0.0094672
ADX	180	0.0005975	0.0086469
AXJO	230	0.0006648	0.0069479
CAC40	231	0.0007271	0.0079969
DJIA	230	0.0005609	0.0072776
FTMIB	228	0.0007997	0.0089854
FTSE100	229	0.0002862	0.0070348
GDAXI	227	0.0007878	0.0083612
GSPTSE	228	0.0004087	0.0043428
HSI	225	0.000131	0.0097464
IMOEX.ME	228	0.0011242	0.0069037
JKSE	222	−0.0001297	0.0072075
KLSE	224	−0.0003385	0.0049151
KOSPI	225	0.0000722	0.0078631
N225	218	0.0005636	0.008545
NSEI	220	0.0006775	0.0089491
SET50	221	−0.0000365	0.0070016
SSEC	225	0.0005439	0.0114002
STI	234	0.0000212	0.0059575
SZCS	225	0.0010874	0.0145107
TPE TAIEX	227	0.000739	0.0064854
Panel B: Post-event period from 1-20-2020 to 3-18-2020			
AAXJ	41	−0.0075615	0.0329298
ADX	35	−0.0083242	0.027362
AXJO	42	−0.0080026	0.0287038
CAC40	43	−0.0108071	0.0284906
DJIA	41	−0.0086318	0.0399845
FTMIB	43	−0.0100961	0.0372304
FTSE100	43	−0.009224	0.0251664
GDAXI	43	−0.0105135	0.0275578
GSPTSE	42	−0.0088958	0.0366424
HSI	41	−0.0062992	0.0170684
IMOEX.ME	41	−0.0097904	0.0227542
JKSE	43	−0.0084753	0.0186309
KLSE	42	−0.0059079	0.0141128
KOSPI	41	−0.0082426	0.0189679
N225	41	−0.0086462	0.0180879
NSEI	41	−0.0082748	0.023342
SET50	42	−0.0100601	0.0302894
SSEC	37	−0.003025	0.0201833
STI	43	−0.006874	0.0158945
SZCS	37	−0.001663	0.0254438
TPE TAIEX	35	–0.0075659	0.0175156

**Table 4:** Abnormal return on event day and one day after.

Index	Abnormal Return	
	Event Day	1 Day after Event Day
ADX	0.0068259	−0.0056369
CAC40	−0.003114	0.0003379
GDAXI	0.0019976	0.0062665
DJIA		−0.0000451
FTSE100	−0.002304	−0.0000359
KLSE	−0.0040547	0.0001122
JKSE	−0.006961	0.0007234
KOSPI	0.0046693	−0.0084402
IMOEX.ME	0.0061993	−0.0024564
N225	0.0007641	−0.0080299
AXJO	0.0018052	−0.0009313
GSPTSE	0.002124	0.0006704
STI	−0.0001791	−0.0085767
TPE TAIEX	0.0013035	−0.0577211
AAXJ		−0.0218231
SET50	−0.0078305	−0.0059545
HSI	−0.008803	−0.0244252
SSEC	0.0068395	−0.0119402
SZCS	0.0125077	−0.0118903
FTMIB	−0.0053448	−0.0010451
NSEI	–0.0113759	–0.0042944

**Table 5:** Cumulative abnormal return in the event window (0, 6).

Index	CAR_i_ (0, 6)	*t*-Test	*p*-Value
HSI	−0.0792506 **	−1.975444	0.0482178
KLSE	−0.0258508 *	−1.874593	0.0608488
AAXJ	−0.0443531 *	−1.786196	0.0740676
GSPTSE	0.0065514 *	1.93884	0.0525208
N225	−0.0317926 *	−1.664644	0.0959838
SET50	−0.0446717 *	−1.836844	0.066233
GDAXI	0.0135345	0.5680507	0.5700005
AXJO	−0.00571	−0.2690441	0.7878957
KOSPI	−0.0482862	−1.178526	0.2385869
FTSE100	0.001566	0.079654	0.9365125
IMOEX.ME	−0.0243261	−1.093912	0.2739936
FTMIB	0.0230502	0.8349835	0.4037271
SZCS	−0.0796273	−0.7984056	0.4246351
JKSE	−0.0192188	−1.312946	0.1892012
NSEI	−0.0255788	−1.424322	0.1543534
ADX	−0.001587	−0.0890671	0.9290286
SSEC	−0.0910787	−1.095587	0.2732597
TPE TAIEX	−0.0373395	−0.56546	0.5717609
CAC40	−0.0003477	−0.0180654	0.9855867
DJIA	0.0035989	0.7588902	0.4479183
STI	–0.0234682	–1.011555	0.3117507

Notes: * Significant at the 10% level. ** Significant at the 5% level.

**Table 6:** Cumulative abnormal return in the event window (7, 13).

Index	CAR_i_ (7, 13)	*t*-Test	*p*-Value
ADX	−0.021254 **	−2.327517	0.0199378
SSEC	0.0311078 *	1.890039	0.0587528
SZCS	0.0506241 *	1.741973	0.0815132
AAXJ	0.0022715	0.0942906	0.9248784
HSI	0.0167401	0.6628569	0.5074222
FTSE100	−0.0084192	−0.8061661	0.4201471
IMOEX.ME	−0.0191139	−1.506789	0.1318648
NSEI	0.020922	0.9712772	0.3314103
JKSE	−0.0221123	−1.068213	0.2854244
SET50	0.0136898	0.6219259	0.5339906
AXJO	−0.006852	−0.3604153	0.7185366
CAC40	0.003732	0.2948792	0.7680862
FTMIB	0.0037601	0.1884156	0.8505509
DJIA	0.0088673	0.5705396	0.5683118
STI	0.0117588	0.5398692	0.5892872
GDAXI	0.0012289	0.1095778	0.9127442
KOSPI	0.0105887	0.3394943	0.7342374
GSPTSE	0.0062839	0.9443354	0.3449982
KLSE	0.0028516	0.1731493	0.862534
N225	0.0140107	0.3945872	0.6931475
TPE TAIEX	–0.0073383	–0.4206511	0.6740099

Notes: * Significant at the 10% level. ** Significant at the 5% level.

**Table 7:** Cumulative abnormal return in the event window (14, 20).

Index	CAR_i_ (14, 20)	*t*-Test	*p*-Value
SZCS	0.1079927 ***	3.284576	0.0010214
N225	−0.0337579 **	−2.275619	0.0228688
SSEC	0.0641881 **	2.426681	0.0152377
FTMIB	0.0207867 *	1.679509	0.0930529
ADX	−0.0356547 *	−1.882657	0.0597468
FTSE100	−0.0112175	−0.8909222	0.3729709
JKSE	−0.0189848	−1.443648	0.148838
IMOEX.ME	−0.0053375	−0.3103923	0.7562627
AAXJ	0.0020406	0.1118767	0.9109212
KLSE	−0.0104853	−0.7950887	0.4265619
NSEI	−0.0165062	−1.240968	0.2146177
KOSPI	−0.0055114	−0.3353011	0.737398
GSPTSE	0.0048234	0.870159	0.3842134
HSI	0.0089929	0.5463076	0.5848545
AXJO	0.0081514	1.198896	0.2305684
DJIA	−0.0027707	−0.3628692	0.7167026
CAC40	0.0040015	0.364354	0.7155937
GDAXI	0.0095633	1.116724	0.2641122
SET50	−0.0091309	−0.4958354	0.6200106
STI	−0.0067322	−0.3105193	0.7561661
TPE TAIEX	–0.0200302	–1.145352	0.2520634

Notes: * Significant at the 10% level. ** Significant at the 5% level. *** Significant at the 1% level.

**Table 8:** Cumulative abnormal return in the event window (21, 27).

Index	CAR_i_ (21, 27)	*t*-Test	*p*-Value
FTSE100	0.029436 ***	2.710452	0.0067191
CAC40	0.0241526 *	1.881764	0.059868
KLSE	0.0005445	0.0180252	0.9856187
GSPTSE	−0.0121544	−1.029907	0.3030536
NSEI	−0.0138207	−0.6640217	0.5066764
SSEC	0.0371332	0.7138488	0.4753207
JKSE	−0.0040976	−0.1965423	0.8441858
DJIA	−0.0105779	−1.122875	0.2614908
TPE TAIEX	−0.0250806	−0.6890613	0.4907847
ADX	−0.0222626	−0.5759524	0.5646474
N225	−0.0556228	−1.572033	0.115943
GDAXI	0.0195479	1.425256	0.154083
IMOEX.ME	−0.036838	−1.047606	0.2948203
KOSPI	−0.0407597	−0.9009348	0.367623
AXJO	−0.0373936	−1.461306	0.1439314
AAXJ	0.057032	1.603071	0.108919
STI	−0.0062721	−0.3232006	0.7465433
SET50	−0.0208295	−0.2719478	0.7856622
HSI	0.0473891	1.203362	0.2288363
FTMIB	0.0213244	0.5655288	0.5717142
SZCS	0.0027614	0.0391268	0.9687893

Notes: * Significant at the 10% level. *** Significant at the 1% level.

**Table 9:** Cumulative abnormal return in the event window (28, 34).

Index	CAR_i_ (28, 34)	*t*-Test	*p*-Value
TPE TAIEX	−0.1419123 ***	−3.664365	0.000248
NSEI	−0.0873618 *	−1.798962	0.0720247
AXJO	−0.1167886 *	−1.893935	0.0582336
ADX	−0.1858343	−1.353782	0.1758059
JKSE	−0.0139353	−0.250492	0.8022069
CAC40	−0.0373533	−0.7463557	0.4554526
AAXJ	0.0505535	1.008108	0.3134028
SZCS	−0.0345424	−0.839241	0.4013341
FTSE100	−0.0278968	−0.7244245	0.4688052
SET50	−0.0710598	−0.8344743	0.4040138
IMOEX.ME	−0.1615433	−1.63697	0.1016367
STI	−0.0350006	−1.032785	0.3017044
N225	−0.0467718	−1.409184	0.1587808
SSEC	0.0174407	0.3435947	0.7311511
KOSPI	0.0083866	0.2428905	0.8080902
FTMIB	−0.0572362	−0.9230368	0.3559881
GDAXI	−0.0366837	−0.6212927	0.534407
DJIA	0.0278418	0.7289439	0.466036
KLSE	−0.0362832	−1.060241	0.289035
HSI	0.0095766	0.1674873	0.8669866
GSPTSE	–0.0940138	–1.412604	0.1577721

Notes: * Significant at the 10% level. ** Significant at the 5% level. *** Significant at the 1% level.

**Table 10:** Daily cumulative average abnormal return across all indices.

Event Window	Coef.	Se	*t*-Test	*p*-Value
0	−0.0002348	0.00129068	−0.18192307	0.8556431
1	−0.00809842 *	0.00315944	−2.5632496	0.01036975
2	−0.00715138 *	0.00294243	−2.430432	0.01508084
3	−0.0130106 **	0.00357785	−3.6364307	0.00027644
4	−0.01848152 *	0.00748873	−2.4679127	0.01359035
5	−0.02472998 **	0.00691317	−3.5772281	0.00034726
6	−0.02543742 **	0.00684348	−3.7170292	0.00020158
7	−0.02182337 **	0.00630466	−3.4614666	0.00053724
8	−0.02577939 ***	0.00598241	−4.3091966	0.00001638
9	−0.02698855 ***	0.00547445	−4.9299134	0.0000008227
10	−0.02791188 ***	0.00580662	−4.8069029	0.000001533
11	−0.02256519 ***	0.0048827	−4.6214559	0.000003811
12	−0.02026957 ***	0.00519989	−3.8980742	0.00009696
13	−0.02003991 **	0.00548889	−3.650998	0.00026122
14	−0.01792733 **	0.00549579	−3.2620101	0.00110625
15	−0.01904359 **	0.00555927	−3.4255525	0.00061355
16	−0.0182333 **	0.00542373	−3.3617668	0.00077445
17	−0.01529506 *	0.00579366	−2.639966	0.00829143
18	−0.01578378 *	0.00646971	−2.4396415	0.01470184
19	−0.01696656 *	0.00732636	−2.3158232	0.02056792
20	−0.01744843	0.00840386	−2.0762396	0.0378718
21	−0.01903315 *	0.00811395	−2.3457313	0.01898979
22	−0.01782969	0.00924501	−1.9285741	0.05378375
23	−0.02108545 *	0.00778318	−2.709105	0.0067465
24	−0.02158735 *	0.00857419	−2.5177137	0.01181193
25	−0.02095435 *	0.00828558	−2.5290149	0.01143832
26	−0.02219828 *	0.00991002	−2.2399834	0.025092
27	−0.01965741	0.01209225	−1.6256206	0.10403039
28	−0.03025933	0.01530937	−1.9765242	0.04809544
29	−0.03593303 *	0.01453605	−2.4719938	0.01343618
30	−0.04155065 **	0.01311263	−3.1687507	0.00153096
31	−0.03815619 *	0.01552319	−2.4580131	0.01397081
32	−0.04317276 *	0.01727333	−2.4993887	0.01244078
33	−0.0527609 *	0.02012455	−2.6217179	0.00874878
34	–0.0706297 **	0.02192245	–3.2217983	0.00127389

Notes: * Significant at the 5% level. ** Significant at the 1% level. *** Significant at the 0.1% level.

**Table 11:** Summary Statistics.

Variable.	Obs	Mean	Std. Dev.	Min	Max
*AR*	735	−0.0018	0.0137	−0.0938	0.0402
*ReturnM*	735	−0.0046	0.0180	−0.0949	0.0488
*Return*	735	−0.0045	0.0169	−0.1027	0.0509
*Log_case*	735	7.3615	1.0955	4.3944	9.9323
*VIX*	688	23.0242	12.5632	12.85	82.69

**Table 12:** OLS regression results of COVID-19 confirmed cases and stock market indices AR.

	(1)	(2)	(3)	(4)
VARIABLES	*AR*	*AR*	*AR*	*AR*
*Log_case*	−0.00322 **	−0.00179 **	−0.000967 **	−0.000967 **
	(0.00154)	(0.000721)	(0.000406)	(0.000406)
*Return*		0.653 ***	0.863 ***	0.863 ***
		(0.0422)	(0.0239)	(0.0239)
*ReturnM*			−0.456 ***	−0.456 ***
			(0.0287)	(0.0287)
*Asia*				−0.00311 *
				(0.00168)
Constant	0.0117	0.00571 *	0.00325	0.00636 ***
	(0.00717)	(0.00339)	(0.00218)	(0.00233)
				
Observations	735	735	735	735
R-squared	0.174	0.608	0.824	0.824
Year Control	YES	YES	YES	YES
Country Control	YES	YES	YES	YES

Robust standard errors in parentheses. *** *p* < 0.01, ** *p* < 0.05, * *p* < 0.1.

**Table 13:** Mediating effect of volatility index (VIX).

	*Path A*	*Path B*	*Path C*
VARIABLES	*AR*	*VIX*	*AR*
			
*Log_case*	−0.000967 **	2.321147 ***	−0.000326
	(0.000406)	(0.5677434)	(0.000359)
*Return*	0.863 ***		0.867 ***
	(0.0239)		(0.0246)
*ReturnM*	−0.456 ***		−0.498 ***
	(0.0287)		(0.0316)
*VIX*			−0.000222 ***
			(0.0000798)
*Constant*	0.00325	−0.5885524	0.00160
	(0.00218)	(2.97612)	(0.00234)
			
Observations	735	688	688
R-squared	0.824	0.873	0.827
Year Control	YES	YES	YES
Country Control	YES	YES	YES

Robust standard errors in parentheses. *** *p* < 0.01, ** *p* < 0.05, * *p* < 0.1.
